# OBLX: building standardized reference resources for reproducible bioinformatics data analysis

**DOI:** 10.3389/fbinf.2026.1910035

**Published:** 2026-10-07

**Authors:** Luis Kress, Johannes Hausmann, Jonas Freimuth, Barbara Schrörs, Franziska Lang, Jonas Ibn-Salem

**Affiliations:** 1 TRON Translational Oncology at the University Medical Center of the Johannes Gutenberg University Mainz, Mainz, Germany; 2 Faculty of Biology, Johannes Gutenberg University Mainz, Mainz, Germany

**Keywords:** bioinformatics libraries, bioinformatics references, bioinformatics pipeline, cancer genomics, next-generation sequencing, omics processing, reproducibility, standardization

## Abstract

Reproducible next-generation sequencing (NGS) analyses in cancer research require standardized reference genome resources, yet these resources are often assembled inconsistently, limiting the comparability of results across pipelines and studies. While existing solutions support a range of workflows, they provide only limited documentation of the included data and processing steps. Furthermore, some of these resources are not version-matched or outdated, making them less suitable for integrated and reproducible cancer genomics analyses. To address this gap, we developed the Omics builder for Bioinformatics resource Libraries and indeXes (OBLX, https://github.com/TRON-Bioinformatics/oblx/), a fully documented, configurable and reproducible pipeline for building standardized NGS reference libraries. First, the pipeline retrieves genome and transcriptome reference sequences, their corresponding annotations, and population variant resources from public sources. Subsequently, OBLX processes these resources to ensure compatibility with commonly used NGS tools, which includes building several bioinformatic indices. The resulting OBLX library may then be directly used by downstream tools and pipelines. By documenting all resources, operations, and intended use-cases, OBLX improves transparency, reproducibility, and interoperability for NGS data analyses.

## Introduction

The analysis of genomic and transcriptomic variants from next-generation sequencing (NGS) data has become a backbone of cancer research and has enabled studying the complex biology of individual tumors across large scale datasets (The Cancer Genome Atlas Research Network et al., 2013; The ICGC/TCGA Pan-Cancer Analysis of Whole Genomes Consortium et al., 2020). Reproducible and standardized analysis methods are essential for the accurate interpretation of tumor biology from NGS data. Recent advances in workflow languages and community-driven end-to-end pipeline frameworks such as Nextflow (nf-core) (Di Tommaso et al., 2017; Ewels et al., 2020) and Snakemake (Mölder et al., 2025) have substantially improved the accessibility, reproducibility, and portability of bioinformatic analyses to large-scale sequencing cohorts. However, one remaining challenge limiting comparability is the non-standardized assembly of reference resources, including reference genome sequences, gene annotations, and population variant databases. Usually, reference resources are assembled on a per study basis, resulting in non-standardized resource libraries that complicate the comparison of results across studies, institutions and individual tools. This is evident in large-scale efforts such as TCGA (The Cancer Genome Atlas Research Network et al., 2013) and GTEx (Lonsdale et al., 2013), which rely on different reference genomes and annotations. For example, the GTEx pipeline (Aguet et al., 2025) uses a reference genome including ERCC spike-ins but excluding haplotypes, alternate contigs, and decoy contigs, whereas the TCGA pipeline (Grossman et al., 2016) includes these alternative sequences and additionally incorporates viral sequences. Likewise, GTEx uses GENCODE (Mudge et al., 2025) v39, while TCGA provides annotations based on GENCODE v36 and v22. Therefore, the lack of version-matched, up-to-date and self-contained standardized reference data libraries for downstream NGS analysis remains a pervasive challenge in the field.

Several efforts have been made to address this problem. For instance, the Broad Institute’s GATK (van der Auwera and O’Connor, 2020) best practices provide curated resource bundles for somatic variant calling workflows. However, some of the provided resource files are outdated, and detailed documentation on retrieval and processing of the data is missing. To support standardized fusion gene detection and other cancer transcriptomics analyses, the Trinity Cancer Transcriptome Analysis Toolkit (CTAT) introduced the CTAT Genome Lib Builder (Haas et al., 2019), which assembles reference libraries tailored to the specific requirements of tools such as STAR-Fusion (Haas et al., 2019) and Trinity. refgenie (Stolarczyk et al., 2020) enables both the download of pre-built reference genome assets and the construction of custom genome assemblies. Though the capability to manage custom assets alongside standardized reference resources, the provided pre-built assets and asset-building workflows are primarily tailored to read-mapping applications. The nf-core community developed the nf-core/references (Ewels et al., 2020) pipeline that provides Nextflow modules tailored specifically to nf-core universe pipelines such as nf-core/sarek (Hanssen et al., 2024) and nf-core/rna-seq (Patel et al., 2026). The modules can be integrated directly into nf-core pipelines to generate indices on a per-run basis, and the pipeline additionally supports the creation of a standalone, reusable reference library. It supports a broad range of aligners and quantification tools and benefits from the shared nf-core community infrastructure. However, like refgenie, nf-core/references is designed as a general-purpose index builder and currently does not provide resources such as up-to-date gnomAD-derived (Karczewski et al., 2020) germline filtering files, problematic region BED files, cancer-associated viral genomes, or snpEff (Cingolani et al., 2012) databases based on matching GENCODE annotations. As an alternative, nf-core pipelines can retrieve pre-built references from the publicly accessible AWS iGenomes S3 bucket (https://ewels.github.io/AWS-iGenomes/). However, the reference files and indices were generated in 2016 using tools from that era (e.g., STAR v2.5.1b, Bismark v0.14.5) and have not been comprehensively updated since, rendering them increasingly outdated for current best-practice analyses.

Here, we present the Omics builder for Bioinformatics resource Libraries and indeXes (OBLX), a reproducible and configurable Snakemake-based pipeline for the standardized assembly and curation of reference genome resources for NGS analysis. The pipeline downloads several version-matched references and annotation files (e.g., from GENCODE, Ensembl (Dyer et al., 2025), UCSC (Casper et al., 2026), and UniProt (The UniProt Consortium et al., 2025)) and performs transformations needed for accurate variant calling or transcript analysis, such as masking of problematic and pseudoautosomal regions. In addition, the pipeline builds alignment and quantification indices for widely used tools, including BWA (Li and Durbin, 2009), STAR (Dobin et al., 2013), and snpEff (Cingolani et al., 2012), ensuring that the resulting library is immediately applicable across a broad range of bioinformatics workflows. The resulting OBLX library is designed for seamless compatibility with commonly used tools and pipelines such as Salmon (Patro et al., 2017), Sarek (Hanssen et al., 2024) and custom downstream analysis tools such as splice2neo (Lang et al., 2024). Besides the workflow, pre-built OBLX libraries are available for download (https://tron-bioinformatics.github.io/oblx). OBLX and any created OBLX library will enable straightforward and sustainable use of standardized reference data and therefore facilitate consistent, cross-study comparisons in NGS analyses.

## Materials and methods

### Overview of OBLX

To facilitate the reproducible and standardized assembly of references and indices for omics data analysis, we developed the Snakemake-based (Mölder et al., 2025) workflow OBLX. OBLX reproducibly downloads version-matched genome resources and processes those files into formats directly usable by commonly used NGS tools and automatically constructs tool-specific indices (Figure 1; Supplementary Table S1; Supplementary Figure S1). A broad range of bioinformatic tools commonly integrated in standard workflows are supported (Figure 2).

**FIGURE 1:**
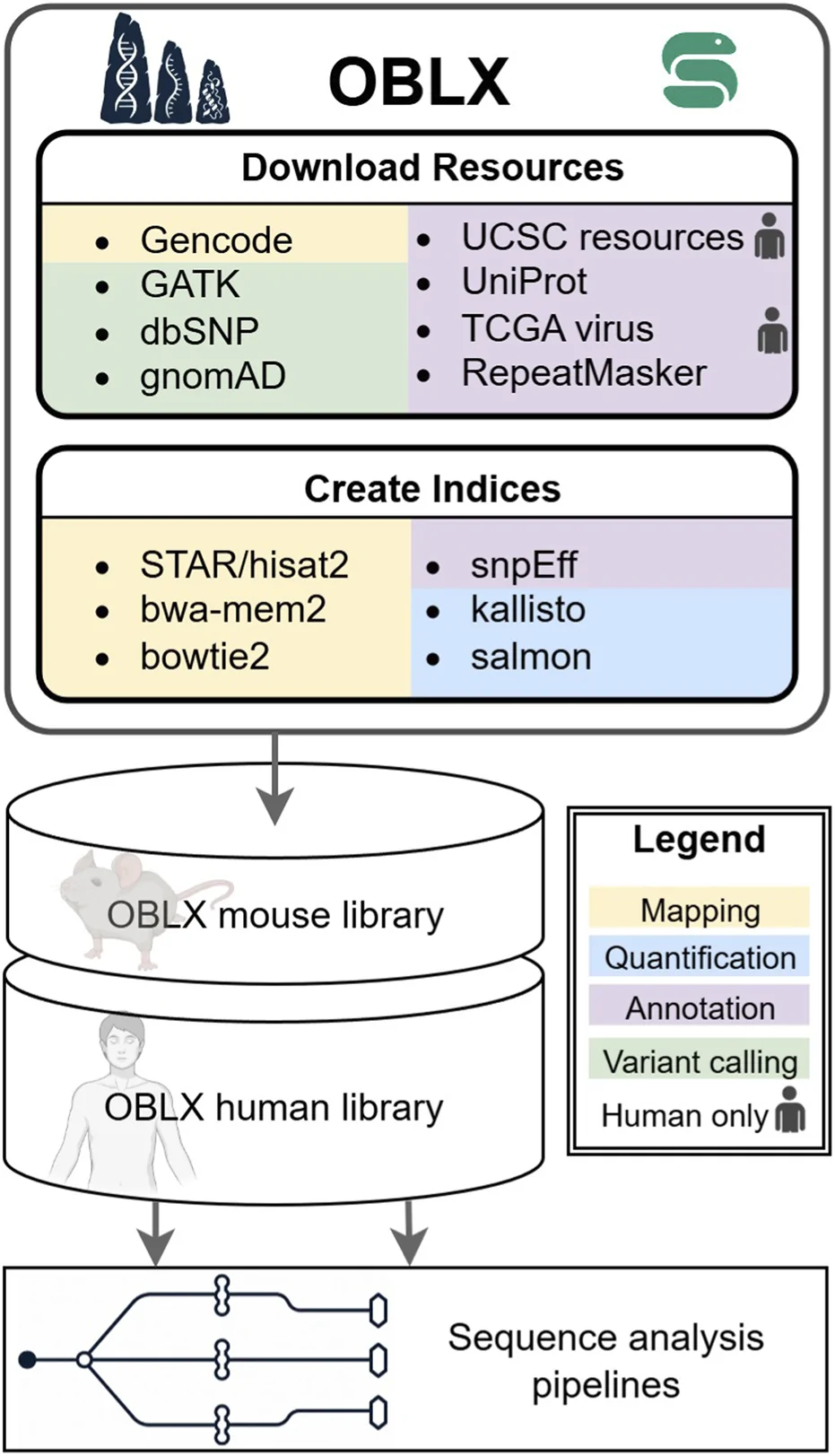
Overview of the OBLX workflow: OBLX consists of two modules: (1) downloading required resource files from online repositories such as GENCODE and UCSC, and (2) using these resources to generate indices and resource files for bioinformatics pipelines and tools. A resulting OBLX library is organism-specific and can be reused across multiple downstream analysis pipelines. Partially created with BioRender. Kress, L. (2026) https://BioRender.com/3h5o7x1.

**FIGURE 2:**
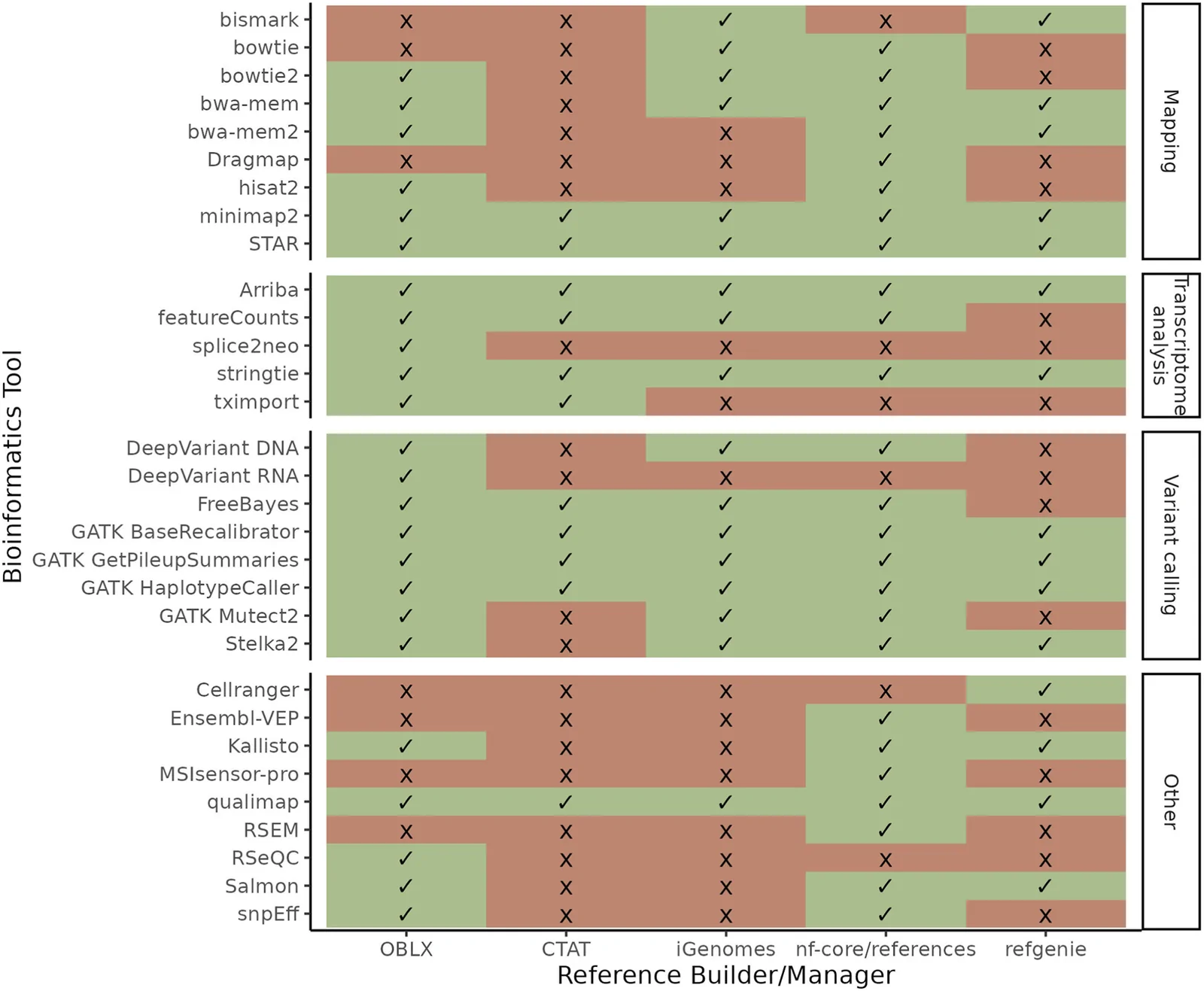
Supported bioinformatics tools (rows, grouped by purpose) of selected reference building/management software (columns). Tools are indicated as supported (✓) if all required mandatory indices/resource files are generated by the reference building/management software.

The workflow is organized into two sequential, independently executable steps: “Download Resources” and “Build Indices”, only requiring the directory to the resulting OBLX library as input. Both steps are fully parameterized through a YAML-based configuration system, enabling user-defined specification of GENCODE (Mudge et al., 2025) release versions, genome builds, gnomAD (Karczewski et al., 2020; Chen et al., 2024) releases, allele frequency cutoffs, generic exome definitions, and aligner-specific parameters, while sensible defaults are provided for immediate use. The workflow supports the construction of human and mouse OBLX libraries, enabling use in clinical as well as pre-clinical analyses. Human libraries additionally include ENCODE mappability (Amemiya et al., 2019) tracks, vendor-specific exome definitions, and gnomAD population frequency resources. Created mouse libraries incorporate Ensembl-based germline variants (Keane et al., 2011; Dyer et al., 2025) with chromosome renaming to match the GENCODE nomenclature, ensuring consistent contig naming across all generated assets.

If any download fails, the workflow resumes per default at the failed process. OBLX supports managing software environments using Conda (The Bioconda Team et al., 2018; Conda Contributors, 2023) or Apptainer (Kurtzer et al., 2017; 2021). It is recommended to use Apptainer to ensure isolation of the computational environment.

Lastly, OBLX comes with extensive online documentation (https://tron-bioinformatics.github.io/oblx), describing the online repositories used for data retrieval and data processing (e.g., which regions in the genome were masked, see GENCODE resources). In the following, we describe the two steps and the resources and indices that are created by OBLX (see also Supplementary Table S1; Supplementary Table S2).

### Download Resources

In the first step “Download Resources”, reference and annotation files are retrieved from the respective online repositories and prepared for downstream index construction. The input for this step is the OBLX library output directory, a config file in YAML format specifying the organism (default: human), GENCODE release (default: 49), genome build (default: GRCh38), and gnomAD release (default: 4.1). The pulled genome resources contain the reference genome, as well as annotation related resources like UniProt (The UniProt Consortium et al., 2025) and RepeatMasker BED (Smit and Hubley, 2026). Additionally, in human mode only, OBLX retrieves germline resources from the GATK bundle, dbSNP (Phan et al., 2025) and gnomAD population-level genomic variant resources, problematic regions bed from UCSC (Casper et al., 2026), and viral sequences.

#### GENCODE resources

OBLX retrieves the GENCODE annotation in gene transfer format (GTF), the primary-assembly reference FASTA, the reference transcript sequences FASTA, and the TrEMBL/SwissProt (The UniProt Consortium et al., 2025) metadata mapping from GENCODE. In human mode, the reference FASTA is hard-masked for Genome Reference Consortium, (n. d.) defined exclusion regions, including false duplications and non-human contamination, as well as pseudoautosomal regions as defined by Ensembl (Dyer et al., 2025) for the human genome. Based on the GENCODE annotation, additional resource files are generated, including reference splice sites, transcript-to-gene mapping, and chromosome size files.

#### GATK bundle

The GATK (van der Auwera and O’Connor, 2020) bundle contains files which are widely used for mutation calling. Those files are only available for human and are therefore only downloaded when creating the human OBLX library. Mills_and_1000G_gold_standard_indels and Homo_sapiens_assembly38.known_indels are downloaded as indel resources and Homo_sapiens_assembly38.dbsnp138, 1000G_phase1.snps.high_confidence.hg38, 1000G_ omni2.5.hg38, and hapmap_3.3.hg38 as SNP resources.

#### Germline variants

In addition to germline variants from the GATK bundle, the “Download Resources” step retrieves germline variants from dbSNP. For the human OBLX library, release 151 is downloaded from NCBI (Sayers et al., 2025); for the mouse OBLX library, variants are downloaded from the Ensembl release matching the configured GENCODE release (as dbSNP is not available for mouse on NCBI), and chromosome names are converted from Ensembl to GENCODE naming scheme. Furthermore, whole exome sequencing (WES) and whole genome sequencing (WGS) germline variants from gnomAD (default: v4.1) are downloaded in human mode. These variants are used to generate up-to-date af_only_gnomad_hg38 resources required by Mutect2, separately for the gnomAD exomes and genomes subsets. Each file contains germline variants for the chromosomes listed in the configurable chrom_filter (default chr1–22, chrX, chrY) with a configurable minimum allele frequency (default: 0.001) and PASS filter status. Here, all annotations are removed and only allele frequency information is retained. Based on gnomAD variants, a common_biallelic_chr1 resource for GATK GetPileupSummaries is generated by retaining only variants on chromosome 1 with allele frequency >0.05, a maximum of two alleles, and PASS filter status.

#### Mappability

Problematic genomic regions are retrieved from the ENCODE “Blacklist” (Amemiya et al., 2019) and the UCSC GRCh38 problematic regions resource. The binary BigBed files are converted to BED6 with bigBedToBed (Kent et al., 2010); the UCSC problematic regions file is additionally trimmed to BED6 to remove comment columns, providing a standardized interval set for overlap-based filtering and downstream artifact removal in short-read sequencing analyses.

#### Viruses

Common cancer-associated viral genomes, including human papillomavirus and Epstein-Barr virus, are obtained based on the strain list defined by TCGA (The Cancer Genome Atlas Research Network et al., 2013). The set of viral strains is provided as a configurable resource file that can be changed or extended according to user requirements. Viral genome sequences are obtained via NCBI efetch (Kans, 2025) and supplied as a multi-FASTA file for downstream analyses.

#### Repeat regions

Interspersed repeats and low-complexity sequences are obtained from the UCSC golden path RepeatMasker annotation dump (rmsk.txt.gz). The resulting “rmsk” output is filtered to the standard chromosomes, converted to BED6 format with the repeat name and Smith-Waterman alignment score retained, and strand-aware merged with bedtools (Quinlan and Hall, 2010) merge subcommand for downstream overlap analysis.

#### UniProt download

UniProt protein annotations are downloaded from the UniProtKB (The UniProt Consortium et al., 2025) FTP archive (https://ftp.uniprot.org/pub/databases/uniprot) to ensure the use of a UniProt release matching the GENCODE/Ensembl version. The correct UniProt version is identified by downloading the Ensembl external_db.txt.gz from https://ftp.ensembl.org/pub/ and parsing the referenced UniProt release (rule extract_uniprot_release_from_ensembl_external_data). Finally, the UniProtKB entries for the respective organism are obtained. A fixed set of fields is extracted from the UniProtKB entries (programmatically_get_uniprot_from_dat.py): protein names, organism name, taxonomy, sequence properties (sequence, length, mass), functional annotations (transmembrane, intramembrane, and topological domain features), Gene Ontology (GO) terms (cellular component, molecular function, biological process), post-translational modification features (MOD_RES), and cross-references to Ensembl genes and KEGG pathways. Each UniProt accession is output as a separate row. To associate the UniProt entries with GENCODE transcript identifiers, the GENCODE-to-SwissProt and GENCODE-to-TrEMBL metadata mapping files, downloaded as part of the GENCODE resource step, are concatenated and left-joined onto the UniProt annotations by UniProt accession. The resulting file (resources/uniprot/uniprot_annotations.tsv) provides a per-transcript mapping to the full set of UniProt annotation fields and is available for both human and mouse genome libraries.

### Build indices

The second step “Build Indices” generates bioinformatics indices for direct integration into bioinformatics analysis and pipelines. Versions of the tools used in OBLX v2.0.2 at the time of writing are listed in Supplementary Table S3.

#### STAR index

Analysis of RNA sequencing often starts with splice-aware read mapping with STAR (Dobin et al., 2013). A STAR index is generated based on the reference FASTA (masked in human mode) and transcript annotation GTF file described in the GENCODE resources section. The parameter --sjdbOverhang specifying the genomic sequence around the annotated junction that is used to construct the splice junction database (https://github.com/alexdobin/STAR/blob/master/doc/STARmanual.pdf) can be defined in the configuration file (default:100).

#### Bwa-mem and bwa-mem2 indices

DNA sequence analysis, including germline and somatic variant calling, is based on alignment of sequencing reads against the reference genome. A bwa-mem (Li, 2013) index is generated using default parameters from the downloaded reference genome (see section GENCODE resources; masked in human mode). BWA-MEM2 (Vasimuddin et al., 2019) is the successor of bwa-mem and produces alignments that are identical to bwa while running approximately 1.3- to 3.1-fold faster (Vasimuddin et al., 2019). OBLX generates a BWA-MEM2 index from the reference genome FASTA file, which is hard-masked in human mode, using default parameters.

#### HISAT2

For splice-aware alignment with HISAT2 (Kim et al., 2019), a Hierarchical Graph FM index is created in human mode with default parameters, incorporating common germline SNPs from dbSNP together with reference splice sites and exons extracted from the GENCODE annotation. A HISAT2 index is currently not produced for mouse because the size of the Ensembl mouse germline SNPs exceeds the index limits.

#### Bowtie2 index

A Bowtie2 (Langmead and Salzberg, 2012) index is generated from the reference genome FASTA file, which is hard masked in human mode, using default parameters.

#### Salmon index

For transcript quantification with Salmon (Patro et al., 2017), a “gentrome” index is generated by adding reference genome chromosomes as decoys to improve expression profiling accuracy. All other index parameters are kept identical to the standard cDNA index. To enable Salmon (Patro et al., 2017) quantification from STAR-generated transcriptome alignments (Aligned.ToTranscriptome.out.bam), cDNA sequences are extracted from the GENCODE GTF file using gffread (Pertea and Pertea, 2020).

#### Kallisto index

In addition to the salmon index, OBLX generates a kallisto (Bray et al., 2016) index for transcript abundance quantification. OBLX uses kbtools (Sullivan et al., 2023) “ref” based on the reference genome and reference annotation (see section GENCODE Resources) in standard mode (--workflow = standard). The included gene types (--include-attribute) can be found in Supplementary Table S4.

#### snpEff database

The pre-built databases distributed with snpEff (Cingolani et al., 2012) are based on older Ensembl releases (v99 and v75). To match the GENCODE-based annotation, OBLX generates a custom snpEff database from the reference genome FASTA and annotation GTF. Database construction is performed in -gtf22 mode using a minimal configuration file including the vertebrate mitochondrial codon table (https://github.com/pcingola/snpeff), together with the -noCheckCds and -noCheckProtein options. The resulting database is named according to the genome build and GENCODE release (e.g., GRCh38.49) and can be used with snpEff by specifying the generated configuration file (see the snpEff section in Supplementary Table S1) via -c, together with -nodownload and the database name.

#### TxDb database

For splice2neo (Lang et al., 2024) and other R and Bioconductor packages, OBLX provides a pre-built TxDb database (Pagès et al., 2026) as well as GenomicFeatures objects (Lawrence et al., 2013) for transcripts, transcripts-by-exons, and transcripts-by-CDS serialized as RDS objects for easy loading in R scripts or interactive R sessions. In addition, a 2bit representation of the reference genome is included to enable fast random access in sequence operations.

### Example bioinformatics analysis with OBLX

We generated an OBLX library using OBLX v2.0.2 for human with default parameters using the following command.

snakemake -s workflow/Snakefile \

--directory </path/to/oblx/library> \

--software-deployment-method apptainer \

--latency-wait 60

A mouse OBLX library was generated using mouse specific parameters.

snakemake -s workflow/Snakefile \

--directory </path/to/oblx/library> \

--software-deployment-method apptainer \

--latency-wait 60 \

--config organism=mouse release=M36 genome_build=GRCm39

Versions of the tools used in OBLX v2.0.2 are listed in Supplementary Table S3.

#### OBLX benchmark

The OBLX pipeline uses the benchmark directive to measure the runtime and memory usage of processes. To benchmark OBLX, human and mouse OBLX libraries were created three times each, using the default memory and thread settings defined in the rule directives of the pipeline code, and computational performance was evaluated across the three replicate runs. These runs were executed on HPC compute nodes (hardware and number of nodes are shown in Supplementary Table S5) using flash storage (NetApp AFF300).

Peak memory consumption was estimated by extracting the maximum “max_rss” value across all benchmark files for the respective run mode (human or mouse). Disk space requirements were determined using the coreutil’s “du” utility (version 9.11). As indication of the individual rule’s runtime, the “s” column of the benchmark table was used. The run time of a whole run was less straightforward to measure as this is not explicitly recorded by Snakemake. Therefore, we used the timestamp of the run’s logfile as the starting time and the timestamp of last modification as reported by the operating system as the stop time. The latter time always lined up within around a minute of the timestamp of the final rule executed.

#### Variant calling with nf-core/sarek and OBLX library

A Nextflow configuration file for nf-core/sarek (Hanssen et al., 2024) was generated based on the assembled human OBLX library using the script utils/write_sarek_config.sh <path/to/created/oblx/library> <nextflow.config.file>. Subsequently, nf-core/sarek v3.8.1 was executed on nf-core testdata for Sarek (samplesheet: https://github.com/nf-core/sarek/blob/b97952e5bac68d5deb93d4a3349a45f146be9830/tests/csv/3.0/fastq_pair.csv) using the previously generated OBLX configuration file (<nextflow.config. file>) with the following command (ran with nextflow version 25.10.4).

nextflow run nf-core/sarek -r 3.8.1 \

-c <nextflow.config.file> \

-profile singularity \

--input samplesheet.csv \

--outdir </path/to/sarek/results>

--tools mutect2,strelka,snpeff \

--only_paired_variant_calling \

--wes \

--intervals </path/to/created/oblx/ library>/resources/exome_definition/ref_ exome.bed

-with-report \

-with-trace \

-with-timeline

#### Transcript quantification with salmon and OBLX library

To obtain the transcript expression, Salmon was run using the following command on test RNA-seq data from the nf-core/rnavar pipeline (samplesheet: https://github.com/nf-core/rnavar/blob/460ef3652925307be767394c5776ba29df92a35b/tests/csv/1.0/fastq_single.csv).

salmon quant \

-i </path/to/oblx/library> -l A \

–1 test_rnaseq_1.fastq.gz \

-2 test_rnaseq_2.fastq.gz \

--validateMappings \

-o transcripts_quant

## Results

### Example application of genome reference library generation

As a proof-of-principle demonstration, we used OBLX to create a genome reference library for human and mouse, hereafter referred to as OBLX libraries. Generating the OBLX libraries required 19.51 ± 3.69 h and 16.24 ± 0.21 h for human and mouse, respectively, on a high-performance computing (HPC) cluster (Supplementary Figure S2A). The effect of the parallelization of the execution of individual rules by Snakemake was assessed by the average total wall clock times taken up by individual rules, resulting in 29.14 ± 3.72 h with 154 rule executions per run for human and 16.94 ± 0.3 h with 39 rule executions per run for mouse (Supplementary Figure S2B). Peak memory usage reached 197.88 ± 0.01 GB during HISAT2 index generation for the human OBLX library and 62.47 ± 0.01 GB during bwa-mem2 index generation for the mouse OBLX library.

The generated OBLX library covered 51 resource files (21 in mouse mode) (Supplementary Table S2) and nine (eight in mouse mode) bioinformatics tool indices, requiring approximately 120.14 ± 0 GB and 81.75 ± 0 GB of disk space for the human and mouse libraries, respectively (Supplementary Figure S2C).

### OBLX reduces genomic asset preparation to two steps

A typical cancer genomic analysis workflow might combine variant calling from WES data with Mutect2 and variant annotation with snpEff as well as transcript quantification with Salmon. To validate the compatibility of an OBLX library with commonly used bioinformatic tools, we called and annotated somatic mutations with the nf-core/sarek pipeline and quantified transcript-level expression with Salmon for nf-core testdatasets. Using nf-core/sarek, we identified 1 and 11 somatic mutations (filter: PASS) with Mutect2 and Strelka2, respectively. We quantified 47 expressed transcripts with Salmon for the nf-core testdataset of the rnavar pipeline. These tools require several genomic resources, including a germline resource such as gnomAD for Mutect2, a reference genome for read alignment, a snpEff database, and a Salmon transcriptome index. While it would have taken several steps to manually retrieve the nine required and eight optional resource files for variant calling and expression quantification, these resources were assembled reproducibly with a single OBLX command (Figure 3). We used an OBLX utility script to create the configuration file for nf-core/sarek using OBLX library outputs, reducing reference preparation to two steps. Thus, OBLX streamlines reference preparation to a simple two-step process.

**FIGURE 3:**
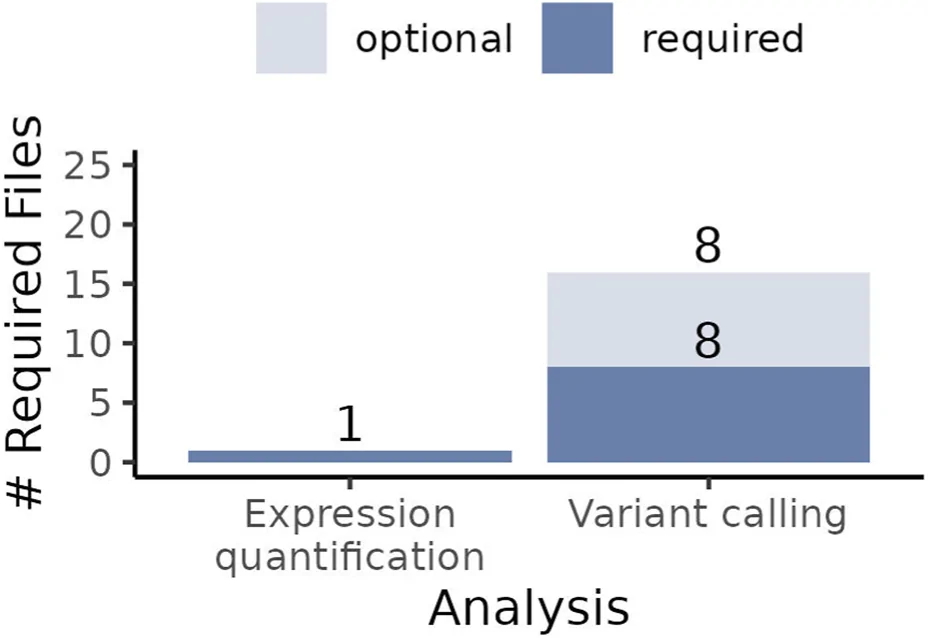
Complexity of preparation of required genome resources and indices for common bioinformatics analyses. Number of unique required and optional files (specified in Supplementary Table S1) on the y-axis for expression quantification analysis (salmon or kallisto) and variant calling (mapping with bwa-mem2, GATK BaseRecalibrator, GATK GetPileupSummaries, GATK Mutect2 and variant effect prediction with snpEff).

## Discussion

Reproducible and interpretable analysis of tumor biology using NGS data requires standardized and documented methods. OBLX provides a well-documented framework for generating omics references and indices. By integrating genome resource download and bioinformatics index generation into a modular workflow, we simplify reference preparation, support the selection of appropriate files for a variety of downstream tools, and enable the creation of consistent and up-to-date resource bundles for analysis. The current implementation supports human and mouse references, making it useful for large-scale analysis of clinical or preclinical NGS datasets, while its modular design allows for easy integration of additional data sources and tools. We showed that the generated OBLX library synergizes well with popular bioinformatics pipelines like nf-core/sarek.

The current implementation of OBLX is primarily designed for short-read WES, WGS, and RNA-seq workflows in cancer research, and does not fully support other high throughput assays such as long-read sequencing or epigenetic assays. Highly specific use cases, such as custom index parameters, hybrid reference genomes, or decoy sequences, should not be covered by a standardized pre-built bundle as they are not suitable for common analyses.

Furthermore, although OBLX does not support all tool versions, genome assemblies, or organisms that may be required for specific workflows, it is designed for straightforward extension. Currently, OBLX supports the GRCh38-based assembly for humans, which benefits from a mature annotation ecosystem including current GENCODE annotations, gnomAD resources, and GATK bundles. While emerging assemblies such as T2T-CHM13 promise improved completeness, their annotation data is not yet sufficiently supported by major providers such as GENCODE. As annotation resources for T2T continue to develop, integration into OBLX may become feasible in future versions. Similarly, organism support is currently restricted to human and mouse, covering most clinical and pre-clinical applications, but the modular design allows extension to further model organisms.

Another practical limitation is the high memory demand of HISAT2 graph-index generation. To support users without access to high-memory infrastructure, we provide pre-built OBLX libraries as a practical alternative (https://tron-bioinformatics.github.io/oblx). This limitation is especially relevant for mouse (mm39), where the Ensembl germline variant resource (European Variation Archive (EVA)-derived SNVs/indels) is comprehensive but heterogeneous across inbred and non-inbred backgrounds, and does not consistently provide metadata for robust variant back-tracing. In our evaluation, the unfiltered mouse variant set caused HISAT2 index generation to fail because of memory constraints. We therefore tested filtering strategies, including selection of multiply observed variants (reducing the set to ∼60%) and exon-restricted variants; however, both approaches still exceeded available memory in our test setup. Consequently, we do not provide a mouse mm39 HISAT2 graph index in the current release and plan to revisit this in a future version.

Other currently available genome resource bundles lack consistent genome resources to match the respective bioinformatics indices and are often outdated. As an example, iGenomes, which is the default option in widely used nf-core pipelines, is based on references that were downloaded in 2016, 10 years ago (https://github.com/ewels/AWS-iGenomes). In contrast, OBLX allows the user to specify the desired release in the input configuration and generates a matched set of reference genome files, annotations, UniProt information, and indices from the same source version. This ensures consistent chromosome naming and gene identifiers across analyses. For example, the snpEff database is generated directly from the selected genome build and annotation, rather than relying on a fixed legacy version such as Ensembl 99. By linking all downstream resources to the same reference release, OBLX improves reproducibility and reduces the risk of mismatched genome resources in subsequent analyses.

Several other tools address aspects of reference genome management for NGS analysis pipelines. Refgenie is a general-purpose genome asset manager that provides an interface for downloading, building, and tracking individual genome assets. While it is well suited for managing and distributing pre-built genome assets across computing environments, it functions primarily as a resource manager. It does not, for example, provide variant calling related resources such as gnomAD-based germline resources for Mutect2 as OBLX does (Figure 2). Nevertheless, refgenie could potentially be used to manage OBLX libraries.

The CTAT Genome Lib Builder assembles reference libraries for the Trinity Cancer Transcriptome Analysis Toolkit and combines resources for fusion gene detection and cancer transcriptomics. However, it is tightly coupled to the CTAT ecosystem and is implemented primarily in custom Perl, which limits extensibility. It does not support DNA-based workflows and does not provide indices for a broad range of general-purpose aligners or variant annotation tools (Figure 2). In addition, documentation of individual data sources, applied transformations, and downstream use cases are limited. This makes it difficult to assess suitability for other analytical contexts or to extend the library to additional tools.

Finally, nf-core/references is a Nextflow-based pipeline for building reference indices used in nf-core pipelines including nf-core/sarek and nf-core/rnaseq. It supports a broad range of aligners and quantification tools and benefits from the nf-core community infrastructure. However, the pipeline relies on user-provided input datasheets with source URLs and does not enforce a standardized resource bundle with documented provenance for each component. In addition, it is designed as a general-purpose index builder and currently does not provide cancer-specific resources such as up-to-date gnomAD-derived germline filtering files, problematic region BED files, cancer-associated viral genomes, or snpEff databases based on matching GENCODE annotations.

While refgenie, CTAT Genome Lib Builder, and nf-core/references each support reference genome management across a range of tools and workflows, they provide limited documentation of data provenance and processing, and do not offer version-matched or up-to-date resource files required for best practices cancer genomics analyses. OBLX addresses these gaps by combining reproducible construction of a comprehensive, self-contained and version-matched reference bundle with full documentation of data provenance, minimal preprocessing, and curated cancer-specific resources such as gnomAD-derived Mutect2 files, problematic region BED files, cancer-associated viral genomes, and GENCODE-matched snpEff indices and UniProt annotations. The resulting OBLX library offers immediate compatibility with both Snakemake and Nextflow analysis pipelines, as well as a broad range of common bioinformatic tools and information on how the library was generated. This allows reproducible analyses of NGS data across a wide variety of bioinformatics workflows which will be more comparable to each other. The modular architecture of OBLX enables future development and will benefit from community contributions to improve portability for cloud-computing, expand organism support, and extend compatibility to additional tools and pipelines.

Taken together, OBLX provides a reproducible, standardized and well documented workflow for automated generation of a consistent genome library from GENCODE releases. The pipeline streamlines genome resource and bioinformatics index preparation for a variety of bioinformatics workflows by reducing manual preprocessing steps, ensuring resource consistency across tools, and improving transparency and traceability of reference generation and modification procedures.

## Data Availability

Pre-built OBLX libraries for human GRCh38 v49 and mouse GRCm39 vM36 are available for download via https://tron-bioinformatics.github.io/oblx. The OBLX software is available on GitHub (https://github.com/TRON-Bioinformatics/oblx) and via the Snakemake workflow catalogue.
