# Supplementary material for: OBLX: building standardized reference resources for reproducible bioinformatics data analysis
Source: Front Bioinform. 2026 Oct 7;6:1910035. doi: 10.3389/fbinf.2026.1910035 (PMC13683796; doi:10.3389/fbinf.2026.1910035)
Supplement: Supplementary file 1 [file Image1.pdf]

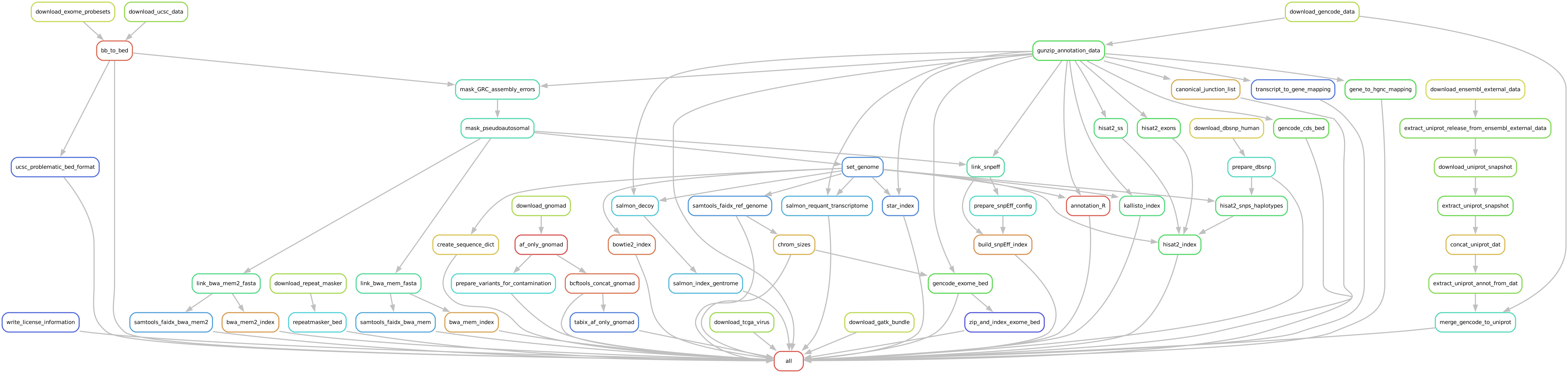
download_exome_probesets
download_ucsc_data
download_gencode_data
bb_to_bed
gunzip_annotation_data
mask_GRC_assembly_errors
canonical_junction_list
transcript_to_gene_mapping
gene_to_hgnc_mapping
download_ensembl_external_data
mask_pseudoautosomal
hisat2_ss
hisat2_exons
download_dbsnp_human
gencode_cds_bed
extract_uniprot_release_from_ensembl_external_data
ucsc_problematic_bed_format
set_genome
link_snpeff
prepare_dbsnp
download_uniprot_snapshot
download_gnomad
salmon_decoy
samtools_faidx_ref_genome
salmon_requant_transcriptome
star_index
prepare_snpEff_config
annotation_R
kallisto_index
hisat2_snps_haplotypes
extract_uniprot_snapshot
create_sequence_dict
af_only_gnomad
bowtie2_index
chrom_sizes
build_snpEff_index
hisat2_index
concat_uniprot_dat
link_bwa_mem2_fasta
download_repeat_masker
link_bwa_mem_fasta
prepare_variants_for_contamination
bcftools_concat_gnomad
salmon_index_gentrome
gencode_exome_bed
extract_uniprot_annot_from_dat
write_license_information
samtools_faidx_bwa_mem2
bwa_mem2_index
repeatmasker_bed
samtools_faidx_bwa_mem
bwa_mem_index
tabix_af_only_gnomad
download_tcga_virus
download_gatk_bundle
zip_and_index_exome_bed
merge_gencode_to_uniprot
all
