# Supplementary material for: OBLX: building standardized reference resources for reproducible bioinformatics data analysis
Source: Front Bioinform. 2026 Oct 7;6:1910035. doi: 10.3389/fbinf.2026.1910035 (PMC13683796; doi:10.3389/fbinf.2026.1910035)
Supplement: Supplementary file 2 [file Image2.pdf]

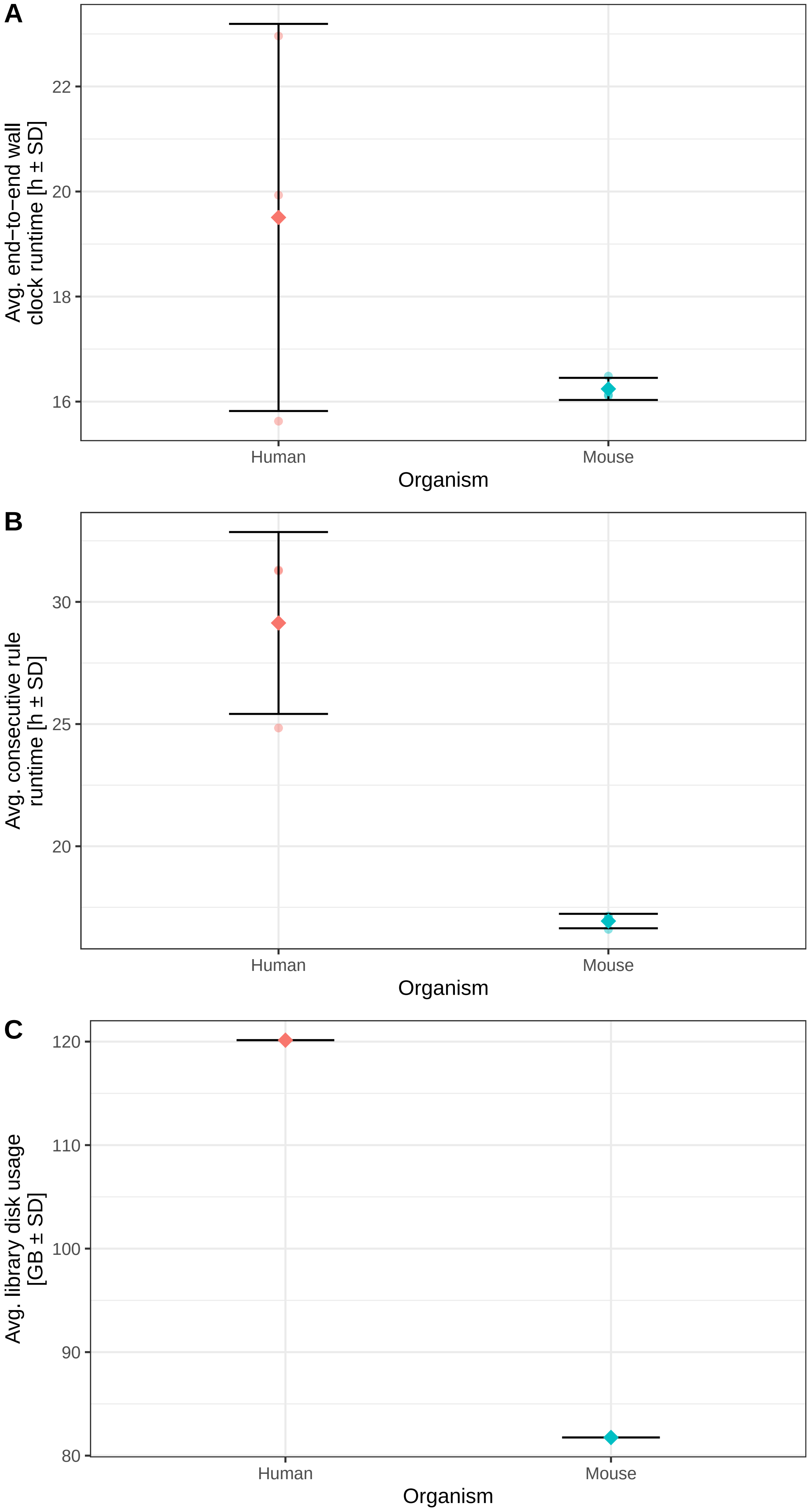
A
Avg. end−to−end wall clock runtime [h ± SD]
22
20
18
16
Human
Mouse
Organism
B
Avg. consecutive rule runtime [h ± SD]
30
25
20
Human
Mouse
Organism
C
Avg. library disk usage [GB ± SD]
120
110
100
90
80
Human
Mouse
Organism
